# The role of Function Words to build syntactic knowledge in French-speaking children

**DOI:** 10.1038/s41598-021-04536-6

**Published:** 2022-01-11

**Authors:** Marie-Thérèse Le Normand, Hung Thai-Van

**Affiliations:** 1 Institut de l’Audition, Institut Pasteur, Inserm, 75012 Paris, France; 2 Université de Paris, Laboratoire de Psychopathologie et Processus de Santé, 92100 Boulogne-Billancourt, France; 3 Université Claude Bernard Lyon 1, 69100 Villeurbanne, France; 4 Service d’Audiologie et d’Explorations Otoneurologiques, Hôpital Edouard Herriot, Hospices Civils de Lyon, 69003 Lyon, France

**Keywords:** Psychology, Human behaviour

## Abstract

The question of how children learn Function Words (FWs) is still a matter of debate among child language researchers. Are early multiword utterances based on lexically specific patterns or rather abstract grammatical relations? In this corpus study, we analyzed FWs having a highly predictable distribution in relation to Mean Length Utterance (MLU) an index of syntactic complexity in a large naturalistic sample of 315 monolingual French children aged 2 to 4 year-old. The data was annotated with a Part Of Speech Tagger (POS-T), belonging to computational tools from CHILDES. While eighteen FWs strongly correlated with MLU expressed either in word or in morpheme, stepwise regression analyses showed that subject pronouns predicted MLU. Factor analysis yielded a bifactor hierarchical model: The first factor loaded sixteen FWs among which eight had a strong developmental weight (third person singular verbs, subject pronouns, articles, auxiliary verbs, prepositions, modals, demonstrative pronouns and plural markers), whereas the second factor loaded complex FWs (possessive verbs and object pronouns). These findings challenge the lexicalist account and support the view that children learn grammatical forms as a complex system based on early instead of late structure building. Children may acquire FWs as combining words and build syntactic knowledge as a complex abstract system which is not innate but learned from multiple word input sentences context. Notably, FWs were found to predict syntactic development and sentence complexity. These results open up new perspectives for clinical assessment and intervention.

## Introduction

The question of how children learn Function Words (FWs) to build syntactic knowledge is a central issue in developmental psycholinguistics and in the applied fields of education, speech-language pathology and early intervention. Since seminal work of Bloom^1^, Brown^2^, Braine^3^, and Maratsos^4^, this issue was modelled in several theories (see Ambridge and Lieven^5^ and Ambridge^6^ for a review). Theories of language acquisition disagree mostly on whether infants come to this process equipped with some language-specific innate predisposition^7–9^ or whether general learning abilities can account for it^10–14^. According to the general learning theories, infants are thought to grasp the statistical regularities and transitional probabilities present in the input they receive, with the help of a critical social learning ability; the outcome of this process of abstraction is semantically specified schemas, related to each kind of meaningful situation. The nativist approach, on the contrary, insists on the innovative linguistic forms and utterances produced by infants, their rule-like patterns of errors and the lack of negative feedback in the input they get.

Leaving aside the nativist vs. emergentist debate, the analyses presented, in this study, are in line with the so-called “syntactic connectivity” account according to which children would learn grammatical words as an interconnected system based on early rather than late structure building (e.g., Ninio^15^).

Herein, we challenge the lexicalist approach or usage-based account, which assumes that children begin to produce multiword utterances before they have any grammatical knowledge (late structure building hypothesis). We postulate that acquisition of FWs and morphemes bootstraps syntactic development (for more detail on the early structure building hypothesis, see Le Normand, Ninio, Szagun^16–18^). According to this latter view, FWs are considered as linguistic cues to the grammatical category of other words. Exploring whether FWs may be grammatically functional early in the acquisition of language is also in line with the bottom-up driven hypothesis. Under this assumption, by attending to the recurring phonological, prosodic and distributional characteristics which FWs typically share young children could derive some useful information for (i) segmenting the continuous speech stream into a set of distinct constituents, (ii) discovering the syntactic class of words and phrases. Young children, accordingly, could be using a comprehensive strategy in sentence processing, one which incorporates not only the meaning-carrying units (i.e., content words) but also the functional elements in language. In the learning process, children extract FWs from the input, making use of the formal distributional properties of their native language. They have a surface knowledge and use FWs to process auditory and linguistic input very early on. Doing so, they gradually construct FWs that manage the multiword sequences in an utterance. Learners do not acquire syntax in a piecemeal and isolated fashion. In such learning-based theories, the young child is sensitive to the phonological, prosodic and distributional patterns in language, and relies on general cognitive (not language-specific) mechanisms to generalize these patterns into a full grammar.

The apparent disagreement between the lexically and grammatically driven approaches (late vs early structure building hypothesis) will be tested in this corpus study using a stringent linguistic coding from the CHILDES^19^.

The main purpose of this study is to investigate how French-speaking children learn FWs to build syntactic knowledge. We hypothezize that acquisition of FWs and morphemes primarily bootstraps syntactic development in order to facilitate syntactic knowledge. A data-driven model will be used to support a syntactic connectivity approach raising the two following questions: (i) Which FW(s) is (are) the most predictive of MLU? and (ii) how FWs are hierarchically organized and reflect sentence complexity. Specifically, we will examine a subset of eighteen FWs since they have a highly predictable distribution from a large corpora of 315 monolingual French children aged 2 to 4.

### Which FW(s) is (are) the most predictive of MLU?

Mean Length Utterance in words (MLU-w) or in morphemes (MLU-m) usually both serve as developmental measure for cross-linguistic comparisons and as a global index of sentence complexity. One important question is to determine whether or not there are subsets of FWs that can predict MLU-w and/or MLU-m. This first question is relevant because FWs include various subcategories which are linguistically and cognitively complex, and acquired at different time periods. Production of noun morphology occurs at an earlier stage of language development than verb morphology. Indeed, it appears that noun morphology (e.g., noun pluralization) is easier to acquire, possibly as a consequence of the semantic and syntactic transparency of nouns. Similarly, determiners are acquired earlier than relative or reflexive pronouns, the latter being linguistically more complex. Determiners are also acquired earlier than time or space prepositions, which are conceptually more elaborate^20^. Thus, by tackling the issue of FWs productivity and complexity, we expected to better understand the processes underlying the grammatical relations (GRs). French-speaking children may acquire the grammatical complex features over a protracted period of time, from 2 to 4 years of age.

Another question raised in this study, is to determine whether MLU should be measured in words (MLU-w) or in morphemes (MLU-m). This matter has consequences, especially when comparing development across languages. Languages differ greatly in how different meanings are mapped into morpho-syntactic structures^21,22^. Some languages appear to be relatively simple with regard to their morphology, while others are viewed as highly complex. MLU-w has been shown to strongly correlate with MLU-m in several languages other than English^23^, including Dutch^24^, Irish^25^, Icelandic^26^, Cypriot-Greek^27^, Eastern Canadian Inuktitut^28^ and Basque^29^. MLU-w has been recommended as an unbiased measure in children who speak dialects or learn multiple languages.

Regarding French, verbs are inflected differently for all persons, (e.g., *il dort, ils dor-ment ‘*he/they sleep’). Ninety-four percent of verbal forms with different inflections are homophonous (e.g., *il chante/ils chantent* ‘he/they sing’)^30^. The gender/number of many nouns/adjectives is made overt only by the presence of the determiner. When audible, these inflections are formed by a vowel (e.g., *chev-al/chev-aux* 'horse/horses') or consonantal marker (e.g., *petit/peti-te* 'small' masculine vs feminine). All these FWs make French-speaking children to control a variety of grammatical morphemes in their early syntax (e.g., gender for determiners or verb agreement for personal pronouns).

### How FWs are hierarchically organized and reflect sentence complexity?

The respective and developmental weight of particular FW should reflect the productivity of frequent grammatical forms and sentence complexity. Here, we attempted to model these two issues using an Exploratory Factor Analysis (EFA). By describing the different FWs factor loadings, it would be possible to draw inferences about the way young children must integrate FWs with a set of multiple syntactic dependencies. First, EFA can be run to search for correlations between FWs. However, because EFA remains an exploratory statistical method to represent the observed data, it does not include any formal a priori test hypothesis. The conclusions that can be drawn from EFA are therefore limited. The degree of independence of the factors identified is thus questionable. Confirmatory factor analysis (CFA), a latent variable approach, can overcome these limitations since it allows comparison between FWs factor loadings. We hypothesize that CFA could define FWs in terms of degree of complexity, thus providing a hierarchical model of the constructs underlying early grammar building. This modeling approach would offer a refined granularity level of difficulty of FWs production compared to existing markers such as MLU. So far, no CFA study has been carried out for estimating the FWs load factors in children aged 2 to 4. Further, CFA should enable prediction of clinical populations performance.

## Methods

This corpus study was approved by Institutional Review Board, from the French National Health Institute, (IRB Number 00000096) and all the research was performed in accordance with their relevant ethical guidelines and regulations. Selection of participants included passing an auditory screening test, scoring in the normal range on an age-appropriate nonverbal cognitive test (Symbolic Play Test^31^) and being a native speaker of French. 315 participants were recruited from homes and nurseries in the Paris area, France. This corpus includes a total of 32,321 utterances, 3016 word types i.e., the total number of different words, and 108,887 word tokens i.e., the total number of words, resulting in a Type/Token ratio of 0.028. Table 1 shows the description of the entire corpora.

**Table 1:** Descriptive summary of the corpora (mean and standard deviation of raw number).

Number of children	Age in months	Total of utterances	Word tokens	Word types	MLU in words	MLU in morphemes
39	24	66 (38)	127 (119)	42.9 (26)	1.60 (.55)	1.86 (.70)
32	27	86 (40)	199 (150)	59.1 (30)	2.11 (.75)	2.50 (.99)
38	30	91 (40)	256 (165)	78.4 (32)	2.52 (.70)	3.02 (.87)
36	33	109 (40)	392 (178)	104 (33)	3.30 (.72)	3.97 (.90)
37	36	112 (46)	414 (234)	107 (36)	3.38 (.90)	4.09 (1.15)
34	39	129 (65)	486 (245)	118 (37)	3.57 (.51)	4.30 (.61)
33	42	116 (69)	494 (368)	123 (51)	3.74 (1.1)	4.49 (1.3)
35	45	113 (58)	491 (317)	124 (44)	3.91 (.65)	4.72 (.80)
31	48	108 (54)	475 (294)	131 (52)	4.01 (.89)	4.82 (1.1)

### Procedure

**Figure 1:**
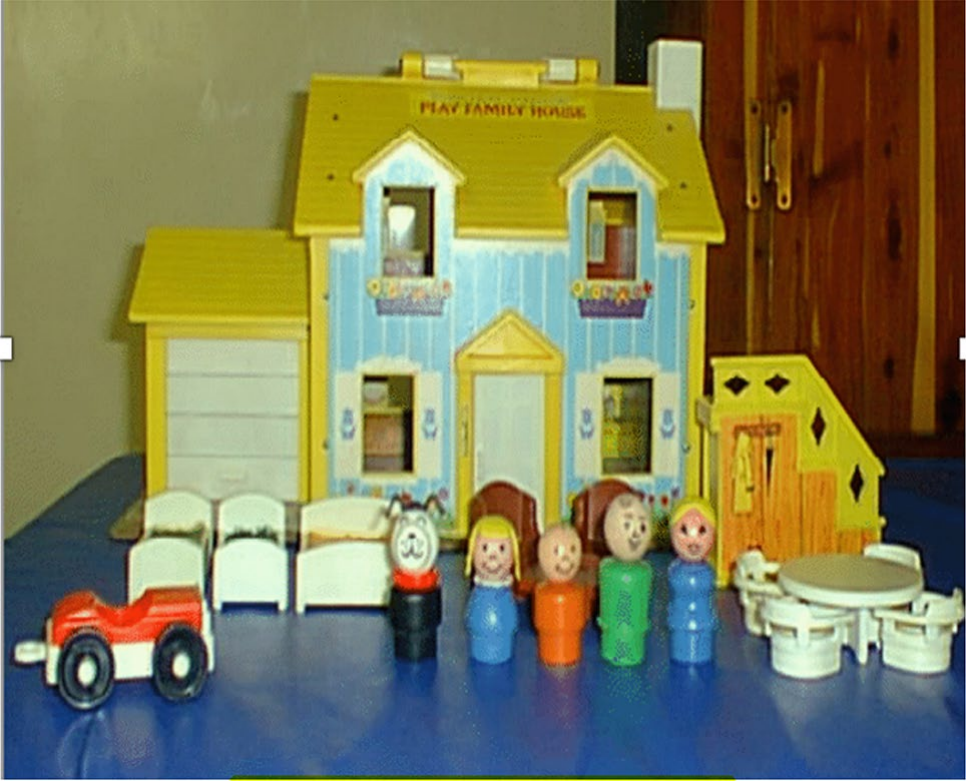
Play situation.

Each child participated in a dyadic interaction with a familiar adult partner (parent or nursery teacher) either in the child's home, nursery or school. Informed written consent was obtained from both parents to videorecorded their child. As children were fairly talkative, we have retained a 20-min sample-time approach. The child and adult were seated at a small table, and the same standardized set of 22 Fisher-Price toys (house, family members, dog, beds, chairs, tables, rocking horse, stroller, cars, staircase) was used with all children (see Fig. 1).

### Transcription and language sample analysis (LSA)

Two trained assistants transcribed the recorded language samples following the transcription and segmentation conventions for spoken French^32–34^, allowing for the computation of linguistic production as described in the corpus processing system CLAN (Child Language Analysis^35,36^. The entire corpus of the children's productions was fully tagged by an automatic part of speech tagger (POS-T). The parser is freely available in the CLAN program which can be found on the CHILDES website (http://childes.psy.cmu.edu). The automated MOR function assigned and disambiguated grammatical descriptions of all the words in these 315 transcripts. Following the running of MOR and POST, we then used the KIDEVAL command to generate spreadsheet output of each child’s language features involving specific morphological features.

The output has the form of this excerpt: *la poupée de Marie qui est dans sa poussette est belle* ‘Mary’s doll in her stroller is pretty’

*CHI: la poupée de Marie qui est dans sa poussette est belle.

%mor:det:art|la&f&sg n|poupée&f prep|de n:prop|Marie

pro:rel|qui v:exist|être&PRES&3 s^v:aux|être&PRES&3s

prep|dans det:poss|sa&f&sg n|poussette&f

v:exist|être&PRES&3 s^v:aux|être&PRES&3 s adj|beau&f&sg = pretty.

Tagging quality was checked by hand, as this corpus is intended to represent a reference for future syntactic analyses of children’s French language corpora. The effective tagging quality of the present corpus after checking by hand averages 97%. From this dataset, a list of 18 FWs has been selected on word tokens (≥ 100 tokens) as described in Supplementary data.

This dataset is available at https://childes.talkbank.org/access/French/MTLN.html.

### Statistical analysis

First, pearson product–moment correlations were performed to examine associations among FWs and MLU-w and MLU-m. Second, a series of multiple regression model was undertaken to estimate whether FWs could predict MLU-w and MLU-m (exact MLU value for each child). Intercept values and regression slopes were determined for every FWs. Finally, Exploratory Factor Analysis (EFA) and Confirmatory Factor Analysis (CFA) were run to extract the factors and validate the factor structure of GMs by performing an oblique rotation. CFA includes multiple indices of fit, which can be used to test models previously developed through EFA conducted on every GM. The Kaiser–Meyer–Olkin (KMO) measurement was calculated to assess the adequacy of the sampling. Ideally, the KMO should be greater than 0.60. We evaluated the fit of a structural equation model (SEM) to determine the degree of adequacy using different adjustment indices: the index square root approximation error of Steiger Lind (RMSEA), the normalized adjustment index (NFI), the Tucker-Lewis index (TLI), the comparative adjustment index (CFI). These indices are interpreted according to critical thresholds. In general, values below 0.05 and 0.08 for the RMSEA are considered to indicate good and acceptable data adequacy respectively. The CFI and TLI should be greater than 0.95. All statistical analyses were performed under JAMOVI^37^ version 1.6. We used Ωnyx, a graphical SEM software for performing maximum likelihood estimation of parameters in models^38^ (http://onyx.brandmaier.de).

## Results

### Relations between MLU (MLU-w and MLU-m) and Function Words

Correlations between age, 18 FWs and MLU-w and MLU-m values was strongly significant (all *p* < 0.001) as shown in Table 2.

**Table 2:** Correlations between age, 18 FWs and MLU-w, MLU-m.

Function Words	MLU-w	MLU-m
Age (month)	0.695	0.688
Past participles	0.501	0.522
Plural markers	0.624	0.626
Stress pronouns	0.523	0.510
Article determiners	0.718	0.699
Possessive determiners	0.531	0.514
Prepositions	0.758	0.743
Object pronouns	0.568	0.566
Reflexive pronouns	0.511	0.508
Relative pronouns	0.521	0.518
Subject pronouns	0.776	0.761
Demonstrative pronouns	0.555	0.552
Interrogative pronouns	0.509	0.495
Specific pronouns y/en	0.506	0.480
Auxiliary verbs	0.672	0.665
Modal verbs	0.701	0.681
Copula	0.495	0.487
Possessive verbs	0.385	0.396
Third person singular	0.713	0.700

### Estimated MLU in words and in morphemes (MLU-w and MLU-m) on every Function Words

Regression analysis on every FWs provided an estimated MLU-w and MLU-m which ranged from 2.09 to 2.98 and from 2.49 to 3.57, respectively. Table 3 sorts the rank order of estimated MLU-w and MLU-m on every FWs.

**Table 3:** Estimated MLU in words and in morphemes on Function Words.

Function Words	MLU-w F (18,314) = 29.58, *p* < .001	MLU-w F (18,314) = 29.58, *p* < .001
Auxiliary verbs	2.09	2.49
Third person singular	2.09	2.50
Article determiners	2.12	2.68
Prepositions	2.15	2.57
Subject pronouns	2.16	2.58
Past participles	2.29	2.68
Modal verbs	2.31	2.78
Demonstrative pronouns	2.37	2.83
Plural markers	2.47	2.93
Stressed pronouns	2.55	3.04
Interrogative pronouns	2.61	3.13
Object pronouns	2.64	3.15
Specific pronoun y/en	2.64	3.18
Relative pronouns	2.66	3.18
Possessive determiners	2.70	3.24
Reflexive pronouns	2.71	3.24
Copula	2.77	3.32
Possessive verbs	2.81	3.35

### Regression Model predicting MLU-w and MLU-m on FWs

Tables 4 and 5 reveal that subject pronouns, a frequent constrained form was particularly found to be the best predictor of MLU-w and MLU-m accounting for 60% and 57% of the unique variance, respectively. For MLU-w, the following covariates were considered but not included: Past participles, Plural markers, Stressed pronouns, Articles, Possessive determiners, Object pronouns, Reflexive pronouns, Relative pronouns, Demonstrative pronouns, Interrogative pronouns, specific pronouns y/en, Auxiliary verbs, Modal verbs, Copula, Possessive verbs, Third person singular. For MLU-m, the following covariates were considered but not included: Past participles, Plural markers, Stressed pronouns, Articles, Possessive determiners, Object pronouns, Reflexive pronouns, Relative pronouns, Demonstrative pronouns, Interrogative pronouns, Specific pronoun y/en, Auxiliary verbs, Modal verbs, Copula, Possessive verbs, Third person singular.

**Table 4:** Regression model predicting MLU-w on Function Words.

Step	Model	β	β (SE)	β	t	*p*
1	(Intercept)	3.098	0.062		49.667	< .001
2	(Intercept)	2.160	0.058		36.956	< .001
	Subject pronouns	0.028	0.001	0.776	21.741	< .001
3	(Intercept)	2.096	0.058		36.188	< .001
	Subject pronouns	0.017	0.002	0.478	6.827	< .001
	Prepositions	0.020	0.004	0.342	4.879	< .001

**Table 5:** Regression model predicting MLU-m on Function Words.

Step	Model	β	β (SE)	β	t	*p*
1	(Intercept)	3.697	0.077		48.270	< .001
2	(Intercept)	2.567	0.074		34.819	< .001
	Subject pronouns	0.018	0.003	0.417	5.401	< .001
	Prepositions	0.024	0.005	0.333	4.631	< .001
	Past participles	0.017	0.008	0.089	1.978	0.049

Tables 6 and 7 describe the relations between the seven different word types of subject personal pronouns and MLU-w /MLU-m. These two contingency tables show that MLU-w and MLU-m significantly increased as young children used more word types (χ^2^ = 299.9, df = 28, *p* < 0.001 and χ^2^ = 363, df = 35, *p* < 0.001, respectively).

**Table 6:** Contingency tables of Subject pronoun according to MLU-w.

MLU-w		Word types								Total
		0	1	2	3	4	5	6	7	
1	N	18	34	15	6	0	0	0	0	73
	Percent	24.7	46.6	20.5	8.2	0	0	0	0	100
2	N	10	11	15	9	17	10	1	0	64
	Percent	1.6	17.2	23.4	14.1	26.6	15.6	1.6	0.0	100
3	N	0	1	4	19	28	31	16	1	100
	Percent	0	1.0	4.0	19.0	28.0	31.0	16.0	1.0	100
4	N	0	0	0	4	11	17	28	7	67
	Percent	0	0	0	6.0	16.4	25.4	41.8	10.4	100
5	N	0	0	0	0	1	1	8	1	11
	Percent	0	0	0.0	0.0	9.1	9.1	72.7	9.1	100
Total	N	19	46	34	38	57	59	53	9	315
	Percent	6.0	14.6	10.8	12.1	18.1	18.7	16.8	2.9	100

**Table 7:** Contingency tables of subject pronoun according to MLU-m.

MLU-m		Word types								Total
		0	1	2	3	4	5	6	7	
1	N	16	24	4	2	0	0	0	0	46
	Percent	34.8	52.2	8.7	4.3	0	0	0	0	100
2	N	3	18	20	8	6	1	0	0	56
	Percent	5.4	32.1	35.7	14.3	10.7	1.8	0	0	100
3	N	0	4	9	13	23	15	2	0	66
	Percent	0	6.1	13.6	19.7	34.8	22.7	3.0	0	100
4	N	0	0	1	14	20	30	20	2	87
	Percent	0	0	1.1	16.1	23.0	34.5	23.0	2.3	100
5	N	0	0	0	1	6	12	23	6	48
	Percent	0	0	0	2.1	12.5	25.0	47.9	12.5	100
6	N	0	0	0	0	2	1	8	1	12
	Percent	0	0	0	0	16.7	8.3	66.7	8.3	100
Total	N	19	46	34	38	57	59	53	9	315
	Percent	6.0	14.6	10.8	12.1	18.1	18.7	16.8	2.9	100

**Figure 2:**
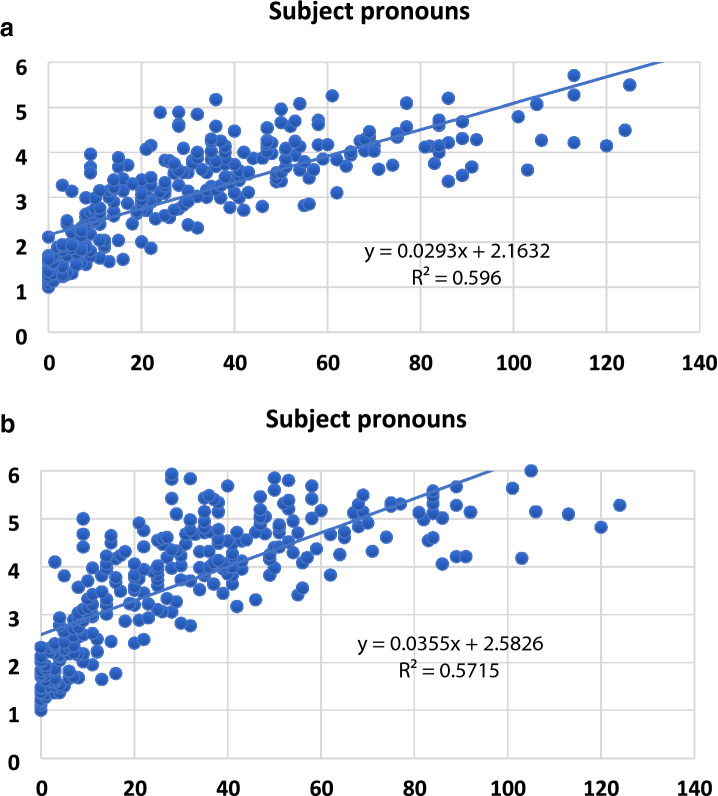
(**a**) Scatterplot between Subject pronouns and MLU-w. (b) Scatterplot between Subject pronouns and MLU-m.

Figures 2a,b illustrate the relations between subject pronouns and MLU-w /MLU-m.

Factor analysis on FWs reflected grammatical productivity and sentence complexity.

**Figure 3:**
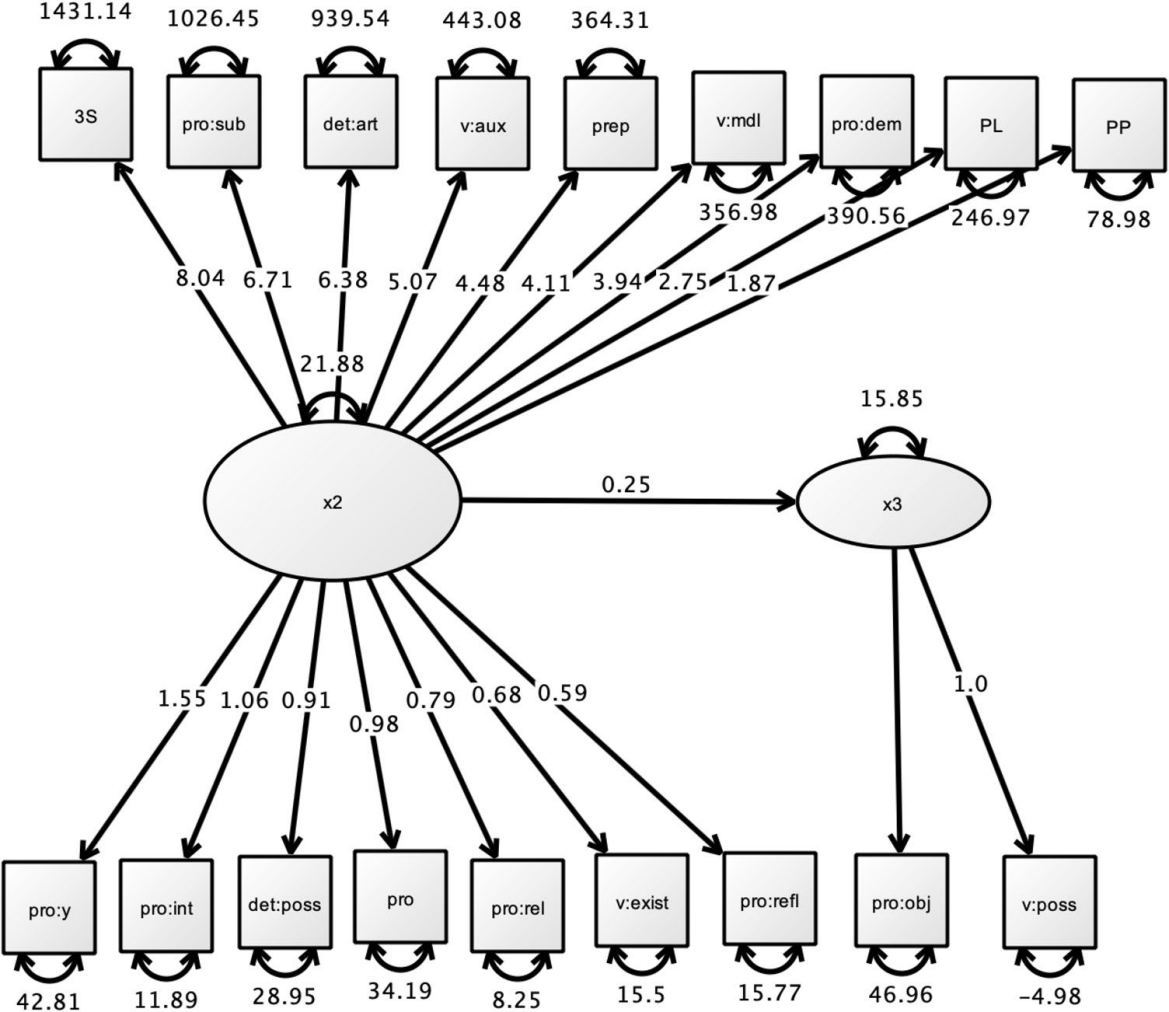
Path diagram among function words from confirmatory factor analysis. Regression relationships between variables are represented by singe-headed arrows whereas variances and covariance relationships are represented by double-headed arrows.

Confirmatory Factor analysis (CFA) yielded a bifactor model: the first factor accounted for 61.44% of the variance (eigenvalue = 11.06) and the second for 6.56% of the variance (eigenvalue = 1.18). A summary of the CFA is presented in Table 8. The first factor loaded on eight FWs of high productivity (third person singular, subject pronouns, articles, auxiliaries, prepositions, modals, demonstratives-pronouns and plural markers) and eight FWs of low productivity (past participles, possessive-determiners, copula, stressed-pronouns and y-pronouns, interrogative-pronouns, relative-pronouns, reflexive-pronouns). The second factor loaded on two FWs of low productivity (object-pronouns and possessive-verbs). This bifactor model showed an excellent adequacy with the observed data (extraction method; maximum likelihood; Rotation method; Oblimin with Kaiser–Meyer–Olkin test normalization (KMO = 0.930), which allows us to formulate the validation of a bifactor model of the 18 FWs. Path diagram using Ωnyx software performed by the maximum likelihood estimation method confirmed the adequacy of the structural equation model (SEM)-Test for exact fit, χ^2^ = 1042—df = 134, *p* < 0.001—Fit measures CFI = 0.844—TLI = 0.822, SRMR = 0.0467—RMSEA = 0.147, (90% CI from 0.138 to 0.155); AIC = 36,049, BIC = 36,256). Path diagram is presented in Fig. 3.

**Table 8:** Confirmatory factor analysis (CFA) across Function Words.

Factor loadings	FWs	β	SE	Z	*p*	Stand. β
Factor 1	Third person singular	31.04	1.324	23.5	< .001	0.967
	Subject pronouns	27.21	1.239	22.0	< .001	0.933
	Articles	24.52	1.178	20.8	< .001	0.905
	Auxiliary verbs	17.99	0.812	22.2	< .001	0.938
	Prepositions	16.94	0.820	20.7	< .001	0.901
	Modal verbs	16.21	0.868	18.7	< .001	0.847
	Demonstrative pronouns	12.73	0.756	16.8	< .001	0.792
	Plural markers	10.64	0.665	16.0	< .001	0.764
	Past participles	6.80	0.457	14.9	< .001	0.726
	Specific pronouns y/en	4.60	0.305	15.1	< .001	0.732
	Interrogative pronouns	3.47	0.236	14.7	< .001	0.718
	Possessive determiners	3.07	0.229	13.5	< .001	0.672
	Stressed pronouns	3.65	0.282	12.9	< .001	0.653
	Relative pronouns	2.35	0.169	13.9	< .001	0.690
	Copula	2.05	0.150	13.6	< .001	0.679
	Reflexive pronouns	1.99	0.176	11.3	< .001	0.585
Factor 2	Object pronouns	5.14	0.285	18.0	< .001	0.954
	Possessive verbs	1.54	0.124	12.4	< .001	0.679

## Discussion

The main purpose of this corpus study was to investigate how French-speaking children learn FWs to build syntactic knowledge. We used a data-driven model to explore in one hand which FW(s) is (are) the most predictive of sentence complexity using Mean Length Utterance expressed either in words or in morphemes (MLU-w and MLU-m), and in the other hand how FWs are hierarchically organized. Similarly to many developmental psycholinguistics, the term of FW was defined taking into account various dimensions of linguistic knowledge including phonology, semantics, syntax, distributional regularity and frequency.

When analyzing a subset of the most frequent FWs i.e., eighteen selected FWs in a large corpus of 315 monolingual French children aged 2 to 4, we challenged the lexicalist view, making the hypothesis that FWs are better connected to syntactic development than Content Words (CWs) since these categories do not contribute to the constitution of the lexicon in the same manner. Nouns and predicates, i.e., CWs, are classes of high density, that strongly contribute to the diversity and enrichment of the lexicon, whereas grammatical words (i.e., FWs) are less diversified classes although being used with greater frequency. FWs learning necessarily involves multiword input and hence it necessitates the mastery of syntactic principles (early structure building hypothesis). CWs, as words possessing semantic content, can be learned from single word utterances, as their meaning is learned from the nonlinguistic context (late structure building hypothesis). Learning FWs starts very early on and consequently is a pre-requisite of syntactic development. FWs, particularly determiners and personal pronouns, do not carry a conceptually complex content. They are easy to learn because they are monosyllabic words prosodically constrained.

One major finding, in this corpus study, was that all eighteen FWs were correlated to MLU-w and MLU-m. Particularly, stepwise regression analyses showed that subject-pronoun was found to be the most consistent predictor of MLU accounting for 60% of the variance in MLU-w and for 57% of the variance in MLU-m. MLU was described here as the standard for measuring children's sentence complexity^39^. This standard has indeed several advantages. First, it is widely used in the field of typical language acquisition all over the world. Second, it captures to a certain extent complexity carried by the number of words in a sentence or the number of morphemes, i.e., inflectional changes on word form. However, MLU is not universally accepted and its reliability has been questioned due to its variability across age-groups^40^. It is therefore important to define the conditions under which MLU is related to age. For instance, MLU has been reported to correlate with age when smaller than 3.5, provided the context of language production is strictly controlled^41^. Other studies also concluded that MLU is highly correlated with the development of morphological and syntactic skills but only until the age of 48 months^42–46^. In our study, a high correlation between MLU-w and MLU-m was found (r = 0.99), indicating that the two can be used indifferently. Both MLU-w and MLU-m were found to correlate with age (r = 0.69).

Another finding from a series of multiple regression analysis showed that productivity of FWs in the children’s earliest multiword utterances was strongly related to MLU ranging from 2.09 to 2.81 for MLU-w, and 2.49 to 3.35 for MLU-m. In the case of determiners, for example, children between age 2 and 4 already produced the full set of determiner system with gender and plural markers to the same extent as adults.

**Figure 4:**
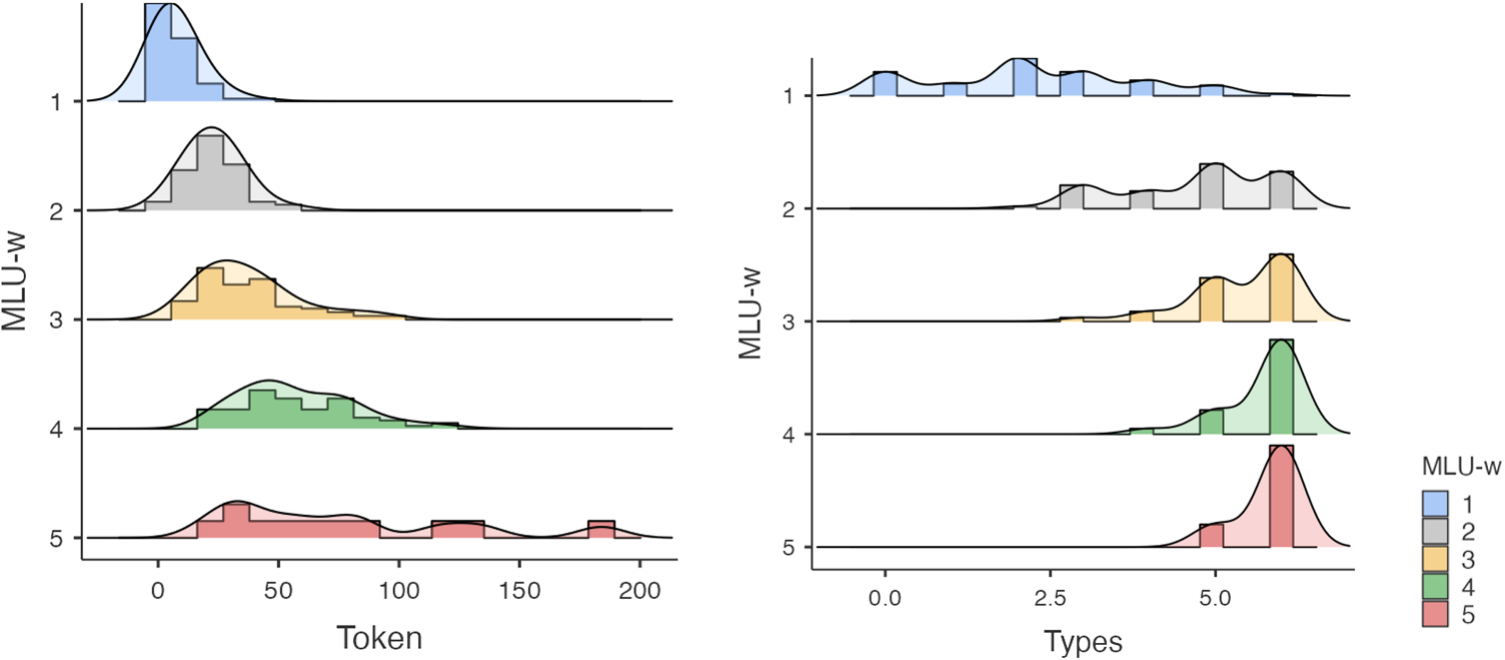
Histogram representing the raw number of word tokens and word types of Article-determiners according to MLU-w *e.g., la/le/un/les/l’/une*. ‘the, a’.

A closer look in Fig. 4, reveals that for MLU-w values ≥ 2.12, six different word types of articles were productively used, despite considerable inter-individual variation: *la,* for 91% of the children; *le,* for 87%; *un,* for 79%; *les* for 70%; l’ for 69%; and *une* for 66%. It should be noticed that at 2 years of age, 36% of children (14 out of 39) omitted determiners in front of noun category with a low MLUw value of 1.60 (SD = 0.55) and MLUm value of 1.86 (SD = 0.70), described as the criterion for identifying the two-word stage and representing the earliest stage of grammatical development^47^). From the age of 1;9 to 2;3 years, children enter a phase of intense development of FWs. This period lasts at least until the age of 3 to 3.5 years. At the end of this phase, the child gives, at least in his most complex productions, the impression of an almost adult language.

**Figure 5:**
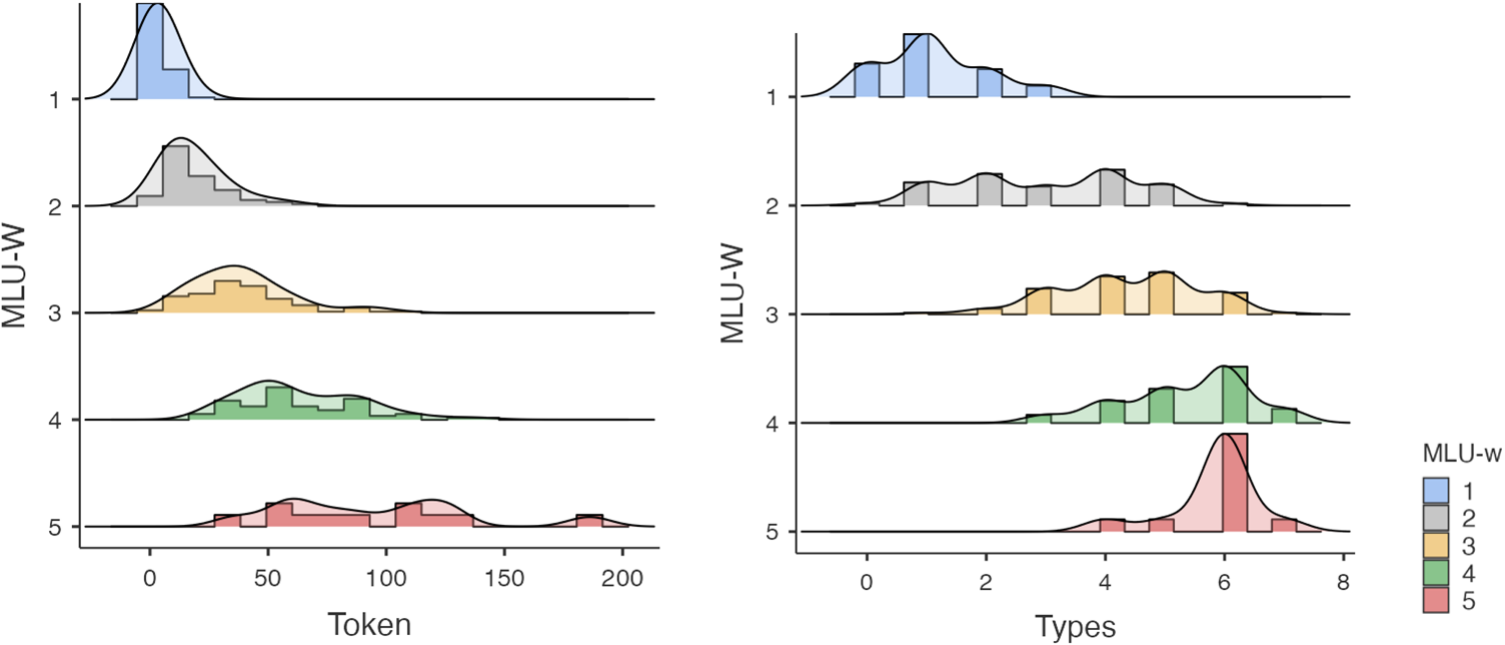
Histogram representing the raw number of word tokens and word types of subject pronouns according to MLU-w *e.g., “il/elle/on/je/tu/ils/elles”(he/she/it/I/you/they-masuline/they-feminine)*.

Most importantly, subject pronouns were found to be the best predictors of MLU-w and MLU-m. Seven word types of subject pronouns were mainly produced according to the following decreasing order: (*il ‘he’* for 91% of the children, *elle ‘she’* for 66% */je ‘I’* for 63% */on ‘it’* for 60% */tu ‘you’* for 47% */ils ‘they’* for 24% */elles ‘they’* for 20%. Similar to the determiner category, considerable inter-individual differences were found in subject personal pronoun category. Young children learn subject pronouns at different paces (see Fig. 5). Some of them learn to inflect words before combining them into larger structures, while others begin to combine words before being able to use morphological markers. Again, such pattern of results supports the early structure building hypothesis related to co-occurrences and generalizations. The young child not only recognizes morphological markers of his native language very early on but also transfers all formal linguistic features from his native language to build and generalize their syntactic knowledge of different pronouns. Word tokens facilitate productivity whereas word types as an index of syntactic diversity make generalization possible.

Although there is great inter-individual variation as sentence length increases, children are sensitive to grammatical categories and FWs from very early on. As soon as their second year, toddlers undergo a two-word stage over a few months and suddenly they begin to construct more and more complex linguistic structures. This means that toddlers, at some point, become able to combine words in a complex, productive, fashion. In our study, the MLU findings support the early structure building hypothesis. Children gradually learn language as a complex system. Being aware of the position and form of FWs, they can use this knowledge to determine the grammatical category of words and their placement.

Regarding to the ongoing debate about how children acquire determiners and use them -i.e., whether early determiner use is lexically^48^ or instead grammatical driven reflecting an underlying abstract grammatical category^49^, results showed that French children recognize and use multiple determiners with gender and plural markers before a noun. This does not reflect an underlying abstract grammatical category but rather an early structure building mechanism at work very early on. Our results also suggest that some prosodic factors and the exposition of infants from birth onwards to the morphological richness of the target language, are involved. As pointed by Demuth^50^, the prosodic form of the particular lexical item and the grammatical morpheme itself interact with each other to determine when a particular function word will appear in children’s speech. For example, French, with phrase-final lengthening, or (iambic) ‘stress’^51,52^, contrasts prosodically with English, which tends to have lexical, trochaic stress^53^.

The same explanation could be given for children’s production of subject pronouns and other pronouns (reflexive pronouns), e.g., the development of some very frequent particular constructions, such as object pronoun “*le, la, les”* (him, her, them), or indirect object “*à lui, à elle”* (to him, to her). As a matter of fact, all these pronouns have specific prosodic forms inherent to the target language. They also have strict obligatory positions, a typical phenomenon of the dependency grammar.

Concerning the extent to which children analyze pronouns or determiners as separate elements, the strong relations found between MLU and determiners and subject pronoun provide developmental evidence that young French children process formal linguistic relationships. Indeed, morphosyntactic markings are associated with a correct gender form, upon the acquisition of determinant-noun and subject pronoun-verb word sequences -e.g., *“la voiture*” (the car) vs. *“le bébé”* (the baby) or pronoun-verb word sequences -e.g., *“il va prendre sa voiture”* (he is go-ing to take his car) vs. *“elle va prendre sa voiture”* (she is go-ing to take her car). All these utterances are mostly correctly marked for gender, number and person.

MLU findings led to the view that there is a dichotomy between the open-class lexicon (i.e., meaning bearing content words like nouns, verbs and adjectives) and the close-class lexicon (i.e., grammatical FWs and inflectional markers like third person singular or plural forms. FWs are learned as combining words whereas CWs are learned as stand-alone lexemes, sometimes as rote-learned utterances—those that always appear in the same form and whose component words are produced neither alone nor in combination with different words. Also, CWs are also learned as intermediate combinations—those whose component words are produced alone and/or in combination with another word without presenting stability in the position occupied by one of the words and presenting a limitation in the number of words that enter into the specific combinatorial pattern. Thus, when English-speaking children use forms like drop it; stop it, make it, they probably use creative forms based upon entrenched schemas which cannot be considered as separate words according to the usage-based account^54^. These creative forms should be differentiated from routines or formulaic expressions, i.e., supra-lexical unanalyzed elements, mostly adverbs such as *s*’*il*-*te*-*plaît* ‘please’, *ça*-*y*-*est* ‘it’s over’ coded as pragmatic words.

The very few fillers found in our study is, however, a relevant phenomenon to support the early rather late structure building hypothesis. Fillers can rarely be interpreted as replacing content words or open-class items. They can be regarded as fulfilling a proto-morphemic role, given the particular context of occurrence, i.e. in the place of functional categories. Fillers or “placeholders” are understood as precursors of functional categories. Furthermore, fillers often have been seen as related to the development of determiners and considered as their possible precursors typically used in a prenominal position. They are phonological approximations of a word and hold the place of FWs. They emerge at one-word utterances and continue to be produced occasionally until word combination takes over from one-word utterances They are reported around 1;8 years in the acquisition of French^55,56^.

Frequent determiners use helps children to discover the GRs of case and gender and to track the relationships between different word types of determiners. The strong impact of determiners on grammatical progress suggests that some generalized knowledge is present in early multiword utterances and drives the construction of grammar indicating a decisive role of determiners in early structure building of FWs.

Even though significant correlations were found between MLU and all 18 FWs, MLU remains a metric too underspecified to reflect GRs of syntactic structures as already suggested by Scarborough^45^. This author created an index of Productive Syntax (IPsyn) obtained from a corpus of 100 utterances, within which 56 specific language structures were identified. This underspecification of MLU has motivated, in our study, the need to refine the multiple regression analysis exploring the respective role of every FW using factor analysis to provide a hierarchical model of early grammar.

The results from the factor analysis also support the view according to which children are thought to be able to use syntactic knowledge very early from a range of categories of their native language. This basic learning in context consisting to learn groups of words rather than single words in different contexts (i.e., the preceding and following words like Article-Noun, Subject-Pronoun-verb or Preposition-Noun) allows the learner to correctly use basic functional categories (e.g., determiners, prepositions, pronouns and auxiliaries) and all morphological markers (e.g., plural and gender for nouns and verbs), and as such plays a crucial role in early structure building. These important findings show that the set of eighteen FWs depends not only on strong relations to the grammatical category of other words but also on their placements and projections. FWs are organized as a complex system.

Structural equation modeling enables to determine loading factors for every FW relative to their degree of complexity: namely, higher-ranking over lower-ranking related to placement and projection. Some functional projections are present as soon as the child produces short multiword utterances. However, other functional projections may be left unspecified, yielding a transitory incomplete grammar. CFA could assign a status to every FW, thus providing a hierarchical model of the constructs underlying non-adjacent dependencies. This modeling approach focuses on the details of linguistic representation in language learners with predictions about what the child must acquire first before starting to produce complex sentences. In our model, the first factor loads sixteen FWs among which eight FWs are strongly weighted: (i) third person singular (ii) subject pronouns, *il/elle/on* ‘he/she/it’ (iii) determiners-articles, (iv) auxiliaries *avoir* et *être* ‘to have/to be’, (v) prepositions *dans* ‘in’*, avec* ‘with’, *pour* ‘for’, (vi) modal verbs *pouvoir* ‘can’ (vii*)*, demonstrative-pronoun *ça* ‘this’*,* and *(*viii*)* plural markers *les* ‘the’. The CFA second factor loads complex forms such as reflexives and object pronouns: e.g., *il/elle se lave* ‘he/she cleans him/herself’, *il/elle le met* ‘he/she puts it’*.*

This hierarchical model of early grammar reflects adjacent and non-adjacent dependencies governing both simple and complex syntactic structures. The concept of dependency stems from traditional grammar of languages. Linguists mostly state that dependencies involve binary relationship between two linguistic units, mostly the governor and the dependent^57–59^. Two restrictions are placed on the dependency structure of a grammatical sentence: first, every word must have a head, and second, every word has only a single head. The exception is the root, namely, the highest word of the sentence, which does not have a head. Although the number of heads per dependent is restricted to one, the converse is not true, so that a head may have a theoretically unlimited number of dependents^60^. Dependencies governing subject-noun/verb agreement and auxiliary/inflectional morpheme relations are acquired earlier than dependencies involving more abstract constituent relationships. In our study, the following GRs were produced by more than 80% of the children. Determiner-noun *la voiture ‘the car’* was produced by 96% of the children, preposition-determiner *dans la* ‘in the’ by 81%, subject-pronoun-auxiliary *il va* ‘he is’ by 89%, subject-pronoun-verb-exist *il est* ‘he is*’* by 92%, demonstrative-pronoun-auxiliary *ça c’est* ‘that is’ by 91%, and auxiliary-main verb *va aller* ‘is go-ing’ by 81%. In contrast, dependencies involving complex GRs such as those found in subject-pronoun-object pronoun *il le* ‘he him’ and possessive verbs were produced by 67% and 54% of the children, respectively.

Statistically, FWs have an extremely high token frequency but a limited set of free and bound morphemes indicating hierarchically organized grammatical relations (GRs) to reflect syntactic knowledge at different times and at different paces. FWs have features which make them easy to learn: they are extremely frequent and have a highly predictable distribution. They are constrained in their distribution due to their placement and projections relative to a sentence context^61^. Between age 2 to 4, French-speaking children productively use the full set of formally marked determiners-articles with correct gender and numbers: e.g., *le, la, les, l’* ‘the’ *un, une, des* ‘a’*.* Similarly, they produce a large variety of prepositions early on *pour* ‘for’, *à* ‘at*’*, *de* ‘from’, *dans* ‘in’, *sur* ‘on’, *avec* ‘with’, and gradually define the case marking of verb argument, allowing the identification of GRs e.g., *all-er à l’école*, ‘go to school’ that constraint word order or introducing an oblique object of the verb e.g., *fin-ir de mang-er* ‘finish eat-ing’ or *donner à Marie* ‘give to Mary’. All these examples indicate congruent GRs within the nominal and/or verbal context. Sentences are marked by GRs: determiners-articles predict nouns while pronouns predict verbs. Words are sorted to form categories such as nouns and verbs and learning regularities over those categories is central to build syntactic knowledge^62,63^.

Our results also support the view that FWs and morphemes bootstrap syntactic development. It has been shown that infants demonstrated robust abilities to abstract both specific and general patterns of varying complexity from auditory and language‐like stimuli^64^. When considering the initial stages of grammar, it is assumed that distributional regularities are readily learned by children and constitute their early generalized syntactic knowledge. According to this statistical learning theory, FWs facilitated infants’ speech segmentation under artificial language learning experiments. For example, 51⁄2- to 8-month-old infants segment speech streams based on adjacent dependencies^65,66^, whereas the ability to compute non-adjacent dependencies seems to develop at around 15 months of age^67^. The constraints on the learnability of non-adjacent dependencies show that statistical learning of these dependencies has greater complexity than statistical learning of adjacent dependencies. Furthermore, FWs occur frequently at the edges of utterances^68–70^, thus enable infants to easily perform word segmentation^71^.

This mechanism of chunking input includes both implicit and explicit distributional learning about the particular form-meaning mapping of the ambient language. If the input from the environment plays an essential role, early grammar building can be viewed as a learning process which starts and evolves in parallel with cognitive development. FWs should be significantly involved in the child’s grammar construction. There is a great deal of evidence in child language literature that processing dependencies proceeds in a highly incremental fashion and can improve our understanding of the child syntactic knowledge. Some FWs are more heavily weighted than others. For instance, development of copula ‘be’ precedes that of possessive verb ‘have’ which in turn outpaces auxiliary ‘do’^72^.

**Figure 6:**
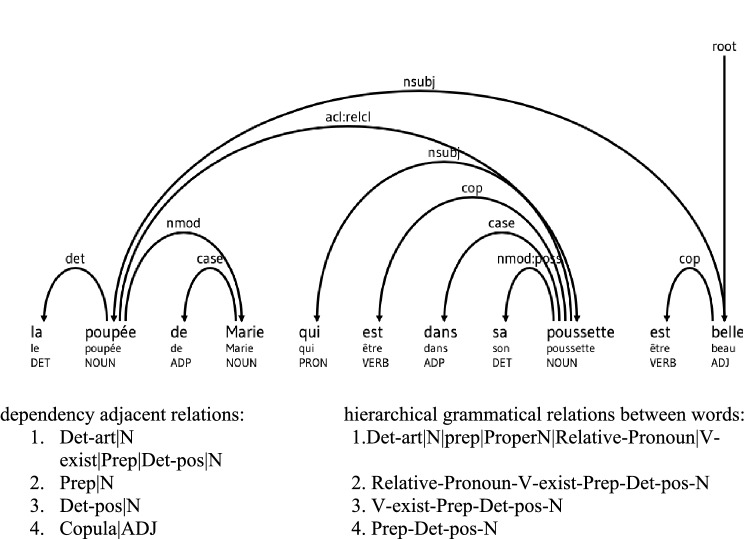
Syntactic tree from dependency grammar (French Meta Grammar http://alpage.inria.fr/frmgdemo)^73^.

A key difficulty is that learning grammar does not involve dependencies just between adjacent but also non-adjacent words. In order to understand the role played by FWs on sentence complexity, computational models based on metagrammar could be directly used to parse sentences and to derive Dependency Grammar (DG). The FRench MetaGrammar (FRMG) PARSER, for instance, is an efficient and accurate solution to cover GR for French. FRMG can parse complex sentences with all FWs and their dependencies in a hierarchically organized syntactic tree (see Fig. 6). In this example, syntactic tree-structure is systematically related to determiner-articles and determiner-possessives, *la|poupée* ‘the|doll’ sa|poussette ‘her|stroller’ prepositions ‘*de|Marie’*, ‘Mary’s|doll’ *dans|sa|poussette* ‘in|her*|*stroller’, copula-be *est|belle* ‘is|pretty’, possessive verb-be *est|dans|sa|poussette* ‘is|in|her|stroller’ and relative-pronoun *qui|est|dans|sa|poussette* ‘who|is|in|her|stroller’.

To further assess learning dependencies across different languages, linguists rely on Universal Dependencies^74^ (UD), a framework for consistent annotation of grammar (parts of speech, morphological features, and syntactic dependencies). UD is an open community effort with over 300 contributors producing nearly 200 treebanks in over 100 languages. (https://universaldependencies.org). An operational grammar like a Tree Adjoining Grammar (TAG) assigns abstract syntactic and semantic representations to an input signal that contains a string of words. It is therefore possible to assign a developmental weight to every particular FW according to DG theory, thus predicting grammar dependencies within complex sentences.

Herein, the results from the factor analysis of eighteen distinctive FWs provided a model of how Typically Developing (TD) French children aged between 2 to 4 years acquire GRs. Critically, to build syntactic knowledge, the child must not only access FWs, but also recognize the crucial roles that FWs play in grammatical organization. French language is an excellent test case to explore this model because FWs show a certain morphological richness. Indeed, French has a diversified and widely used range of FWs of various types. The latter express a variety of distinct meanings, and most of them mark gender, number, and person information (and even verbal flexions for pronouns), resulting in a large variety of FWs within these classes. Determiners, for instance, involve a number of different definite, indefinite and partitive articles marked for gender and number *le*, *la*, *les* (the) *un*, *une* (a).

Importantly, FWs must be learned as combining words and not separated words like CWs. FWs are learned from multiword input sentence context and facilitate syntactic knowledge whereas CWs are learned from single word input.

The results from the factor analysis of eighteen distinctive FWs also provide many possible ways for comparing sentence complexity of FWs. Corpus analysis offers a golden standard showing how groups of FWs hang together. These analyses of GRs from Dependency Grammar are of great value in clinical settings, allowing clinicians and researchers to construct profiles of language by comparing small speech samples collected in a naturalistic context with large corpora. In TD children, clinicians will track syntactic dependencies in sentence structures requiring the productivity of complex FWs such as possessive verbs and object pronouns^75^.

In children with language disorders, the description of the hierarchical organization of FWs is important since the productive use of FWs and morphological markers of gender, number, tense, mood is known to be particularly limited^76–78^. A meta-analysis carried out by Lammertink et al.^79^, indicated a robust difference between children with developmental language disorder (DLD) and those without DLD in their detection of statistical regularities in the auditory input. The detection of statistical regularities is on average, not as effective in DLD compared to TD subjects.

## Conclusions

The present study shows the critical role of FWs to build syntactic knowledge as demonstrated by the strong correlations found between MLU-w, MLU-m and FWs. Indeed, FWs were found to predict syntactic development and sentence complexity. Children may learn FWs based on early rather late structure building. Doing so, they acquire FWs as combining words and build syntax as a complex system which is not innate but learned from multiword input sentences context. This study also indicates that sentence complexity is organized according to a hierarchical model of the most frequent FWs. Clinicians and speech-language pathologists could use such model in their practice. As a matter of fact, model can provide a benchmark for children aged 2 to 4 years upon which various clinical profiles can be analyzed both for diagnosis and interventions purposes. When assessing early grammar, corpus analysis offers a very high degree of ecological validity^80,81^. It supplements standardized appraisal and yields baseline insights into the child’s strengths and weaknesses across language skills. The general parsing techniques have been shown to be effective in the present study. Further research in children with language disorders is needed to better identify atypical syntactic profiles. For instance, clinicians can undertake dependency grammar analyses, which can be harmonized with the UD tagset. Comparisons between TD children and those with language disorders are critical not only for clinical assessment, but also for developing cross-linguistic investigations.

## Supplementary Information

Supplementary Information.

## Data Availability

The datasets generated for this study are available from the CHILDES site at https://childes.talkbank.org/access/French/MTLN.html, Le Normand, M.-T. French MTLN Corpus. https://doi.org/10.21415/T58S3M. (2014). This repository was done in accordance with relevant guidelines and regulations involved for the Protection of Human Subjects from our Institutional Review Board (IRB) and international research ethic committee.
