# Supplementary material for: The role of Function Words to build syntactic knowledge in French-speaking children
Source: Sci Rep. 2022 Jan 11;12:544. doi: 10.1038/s41598-021-04536-6 (PMC8752861; doi:10.1038/s41598-021-04536-6)
Supplement: Supplementary file 1 — Supplementary Information. [file 41598_2021_4536_MOESM1_ESM.pdf]

1. **Supplementary data.**
   Appendix: Coding of Grammatical Categories according to the CHILDES tags with some French examples of Function words

Coding	Grammatical Categories	French examples of Function Words
det	Articles	le la, les, l', un, une (the, a)
det:poss	Possessive determiners	son, sa (his, her)
prep	Prepositions	dans, sur (in, on)
pro	Stressed pronouns	moi, lui, elle (me, him, her)
pro:dem	Demonstrative pronouns	c'est (it is)
pro:int	Interrogative pronouns	où, qu'est ce que (Wh tag)
pro:obj	Object pronouns	le, la, les (him, her, them)
pro:refl	Reflexive pronouns	me, se (myself, him/herself
pro:rel	Relative pronoun	qui, que (who, that)
pro:subj	Subject pronouns	il/elle, ils/ells (he/she,they)
pro:y/en	Specific pronouns-y/en	y'en a (there are)
v:aux	Auxiliary verbs	a pris (has taken), est tombé (has fallen)
v:exist	Copula	est belle (is pretty)
v:mdl	Modal verbs	falloir, pouvoir (must, can)
v:poss	Possessive verbs	a un livre (has a book)
v:pp	Past participles	a pris (has taken)
3rd sg	Third person singular	il dort (he sleeps)
Pl	Nominal Plural forms	les voitures (the cars)

2. Supplementary data . Type/Token distribution of the 18 Function Words

Coding	Grammatical categories	Function Word Type	Function Word Token
		pro:sub|il	5546
		pro:sub|je	1443
		pro:sub|on	1415
		pro:sub|elle	1259
		pro:sub|tu	666
1.pro:sub	subject pronouns	pro:sub|ils	317
		v:aux|être&PRES&3s	4958
		v:aux|avoir&PRES&3s	2129
		v:aux|avoir&PRES&1s	304
		v:aux|être&PRES&3p	295
2. v:aux	auxiliary verbs	v:aux|avoir&PRES&3p	105
		v|dormir-PRES&3s	370
		v|mettre-PRES&3s	361
3.&3s	Irregular-third person singular		
		det:art|la&f&sg	3556
		det:art|le&m&sg	2937
		det:art|les&pl	1186
		det:art|un&m&sg	1151
		det:art|une&f&sg	851
4.det:art	determiner articles	det:art|l'	717
		pro:dem|c'est	3401
		pro:dem|ça	2150
		pro:dem|celui–là	207
5.pro:dem	demonstrative pronouns	pro:dem|celle–là	101
		v:mdl|aller&PRES&3s	2532
		v:mdl|aller&INF	468

		v:mdl|faire-INF	389
		v:mdl|falloir&PRES&3s	347
		v:mdl|aller&PRES&3p	345
		v:mdl|aller&PRES&1s	326
		v:mdl|aller&IMP&2s	285
		v:mdl|pouvoir&PRES&3s	279
		v:mdl|vouloir&PRES&3s	199
		v:mdl|savoir&PRES&12s	181
		v:mdl|aller&PRES&2s	151
6.v:mdl	modals	v:mdl|vouloir&PRES&12s	133
		pro:y|y	1286
7.pro:y/en	specific pronouns	pro:y|en	337
		prep|dans	1259
		prep|de	1161
		prep|à	998
		prep|de&les	737
		prep|pour	581
		prep|à&le	326
		prep|de&le	313
		prep|avec	307
8.prep	prepositions	prep|sur	148
9.pro:refl	reflexive pronouns	pro:refl|se	723
		pro:int|où	686
		pro:int|qui	437
		pro:int|quoi	166
10.pro:int	interrogative pronouns	pro:int|qu'est-ce-que	136

11.v:exist	copula	v:exist|être&PRES&3s	529
		part|faire-PP&m	497
		part|tomber-PP&m	392
		part|fermer-PP&m	150
		part|dire-PP&m	127
		part|voir&PP&m	110
12.PP	past-participles	part|casser-PP&m	101
		pro:obj|le	396
		pro:obj|te	341
13.pro:obj	object-pronouns	pro:obj|me	203
		pro:rel|que	317
		pro:rel|quoi	174
14.pro:rel	relative- pronouns	pro:rel|qui	125
		det:poss|sa&f&sg	282
		det:poss|son&m&sg	191
		det:poss|ma&sg	170
15.det:poss	determiner-possessives	det:poss|mon&m&sg	140
		v:poss|avoir&PRES&2s	236
16.v:poss	possessive-verbs	v:poss|avoir&PRES&3s	141
17.pro	stressed pronouns	pro|toi&sg	205
		n|voiture&f-PL	184
		n|chaise&f-PL	151
		n|bonhomme&m-PL	134
18.PL	nominal plural forms	n|lit&m-PL	100
